# Spatial, temporal, and space-time analysis of leptospirosis cases in Acre, 2001-2022

**DOI:** 10.1590/1980-549720240063

**Published:** 2024-11-16

**Authors:** Leonardo Augusto Kohara Melchior, Kívia Roberta Costa da Silva, Ana Elisa Pereira Silva, Francisco Chiaravalloti-Neto

**Affiliations:** I Universidade Federal do Acre, Postgraduate Program in Health Sciences in Western Amazon - Rio Branco (AC), Brasil.; II Universidade de São Paulo, Faculty of Public Health, Postgraduate Degree in Public Health - São Paulo (SP), Brasil.

**Keywords:** Spatial analysis, Spatiotemporal analysis, Amazonian ecosystem, Leptopira

## Abstract

**Objective:**

To identify clusters of high and low risk for the occurrence of leptospirosis in space and space-time in Acre, between 2001 and 2022, as well as to characterize temporal trends and epidemiological profiles of the disease in the state.

**Methods:**

An ecological study of cases mandatorily reported by health services in Brazil. For the analysis of clusters in space and space-time, the SaTScan software was used, which calculated the relative risks (RR). Additionally, temporal trends were obtained using Prais-Winsten linear regression and epidemiological profiles estimated by incidences by sex and age group.

**Results:**

A high-risk spatial cluster was identified in Rio Branco, Bujari, and Porto Acre (RR=2.94), occurring mainly between 2013 and 2015, according to the space-time cluster (RR=9.51). The municipality of Cruzeiro do Sul also showed a high-risk spatial cluster (RR=1.31). This municipality and contiguous municipalities showed an increasing temporal trend in cases, while the other municipalities in the state showed a stationary temporal trend. The disease mainly affected men between 20 and 59 years old, followed by young people aged 10 to 19 years. However, the RR of leptospirosis in older women was 2.1 times higher than in older men (95%CI 1.6-2.9).

**Conclusion:**

The findings indicated that leptospirosis, although endemic in the state, had a more significant incidence in certain municipalities and years. Therefore, it is necessary to act with greater or lesser intensity in specific locations and periods, both for the prevention and control of the disease.

## INTRODUCTION

Leptospirosis is an acute infectious febrile disease caused by bacteria of the genus *Leptospira* ^1^. It is transmitted directly or indirectly from animals to humans. Infection usually occurs through exposure of the skin and mucous membranes to water contaminated by the urine of infected animals, especially rats^1^. The disease is strongly associated with hydrological events, especially in tropical and subtropical areas with heavy rainfall, as well as in places with poor urbanization and accumulation of garbage^2^.

Although the incidence of leptospirosis in the world is not known precisely, it is estimated that the disease is one of the main causes of morbidity due to zoonoses in the world^2^. In Brazil, between 2001 and 2022, 76,269 cases of the disease were recorded, and it is endemic in all states^3^. The state of Acre stood out during this period, as its incidence (29.67/100,000 inh.) was 3.67 times higher than that of the second state with the highest incidence, Amapá (8.08/100,000 inh.) and 8.46 times higher than that of Brazil (3.51/100,000 inh.)^3^.

Some scientific studies have used scanning techniques to investigate whether cases of a given disease are randomly distributed in space, time and space-time^4-6^. These tools have made it possible to identify clusters of cases, which are critical areas for transmission of the disease. Based on these findings, it is possible to obtain a more in-depth understanding of the disease, which contributes to the implementation of more effective interventions in prevention and control^7,8^. Thus, to better understand the epidemiology of leptospirosis, this study proposed to identify high and low risk clusters for the occurrence of the disease in space and space-time in the state of Acre, between 2001 and 2022. In addition, we sought to characterize the temporal trends and epidemiological profiles of leptospirosis in the state.

## METHODS

We conducted an ecological study that used Acre as the study area. Acre is a state located in the north of Brazil, in the southwestern part of the Western Amazon^9^. It has an area of 164,173 km^2^ and an estimated population of 830,018 inh., with a human development index (HDI) of 0.719. The state's economy is based mainly on the primary sector, with one of the lowest gross domestic products (GDP) in Brazil^9^.

Acre is covered by the Amazon rainforest and has a warm and very humid climate of the Köppen Af and Am types. Average monthly temperatures vary between 24 and 27°C and annual rainfall reaches approximately 2,100 mm. The state has a rainy season between November and April and a dry season between May and October^10^.

For the study, the spatial units were the municipalities of Acre, and the temporal units were the years 2001 to 2022 and the months from January to December.

This study analyzed the autochthonous and confirmed cases of leptospirosis in Acre, between 2001 and 2022. Confirmed cases were those that met the criteria for clinical-laboratory confirmation (positive test for the disease) or clinical-epidemiological confirmation (suggestive epidemiological history in the 30 days prior to the onset of symptoms, such as exposure to floods, sewage, garbage and high-risk occupational activities and presence in a high-risk area for leptospirosis).

These data were recorded by the Diseases Information System (SINAN) and made available electronically by the Department of Informatics of the Unified Health System (DATASUS), Ministry of Health, Brazil^3^. Cases were extracted by municipality, sex (male and female) and age group (0 to 9 years, 10 to 19 years, 20 to 59 years and 60 years or older).

Demographic data from 2001 to 2022 were obtained from the "Study of population estimates by municipality, sex and age, 2000 to 2022", also made available by DATASUS. These data were extracted for the state of Acre by sex, age group and municipality^3^.

The annual incidence rates of leptospirosis were calculated by the ratio between the number of cases and the population of the municipality, stratified by sex and age group and multiplied by 100,000 inh. The stratification seeks to understand the influence of variations by sex and age group in the population, among the municipalities. Relative risk (RR) was calculated by the ratio between the incidences.

To facilitate the understanding of the epidemiological profile of leptospirosis by age group and sex, the municipalities were grouped into geographic microregions, that is, contiguous municipalities that present specificities. Thus, the 22 municipalities were grouped into five microregions: Rio Branco (Acrelândia, Bujari, Capixaba, Plácido de Castro, Porto Acre, Rio Branco and Senador Guiomard), Sena Madureira (Manoel Urbano, Santa Rosa do Purus and Sena Madureira), Brasiléia (Assis Brasil, Brasiléia, Epitaciolândia and Xapuri), Cruzeiro do Sul (Cruzeiro do Sul, Mâncio Lima, Marechal Thaumaturgo, Porto Walter and Rodrigues Alves) and Tarauacá (Feijó, Jordão and Tarauacá)^9^.

To identify high and low risk clusters of leptospirosis in space and space-time in the state of Acre, purely spatial and spatiotemporal statistics were used with the SaTScan software, version 10.1.1 (http://www.satscan.org/)^11^. Since these are case counts, the discrete Poisson model was used^11^.

Spatial analysis is used to identify high or low risk clusters in space. This analysis does not consider the time factor. Spatiotemporal analysis, on the other hand, considers not only space, but also time as a whole. The parameters for running the tests are similar, only requiring the SaTScan to be informed of the test to be performed^3^.

The annual incidence rates of leptospirosis were calculated by the ratio between the number of cases and the population of the municipality, stratified by sex and age group and multiplied by 100,000 inh. The stratification seeks to understand the influence of variations by sex and age group in the population, among the municipalities. The RR was calculated by the ratio between the incidences.

To facilitate the understanding of the epidemiological profile of leptospirosis by age group and sex, the municipalities were grouped into geographic microregions, that is, contiguous municipalities that present specificities. Thus, the 22 municipalities were grouped into five microregions: Rio Branco (Acrelândia, Bujari, Capixaba, Plácido de Castro, Porto Acre, Rio Branco and Senador Guiomard), Sena Madureira (Manoel Urbano, Santa Rosa do Purus and Sena Madureira), Brasiléia (Assis Brasil, Brasiléia, Epitaciolândia and Xapuri), Cruzeiro do Sul (Cruzeiro do Sul, Mâncio Lima, Marechal Thaumaturgo, Porto Walter and Rodrigues Alves) and Tarauacá (Feijó, Jordão and Tarauacá)^9^.

To identify high and low risk clusters of leptospirosis in space and space-time in the state of Acre, purely spatial and spatiotemporal statistics were used using the SaTScan software, version 10.1.1 (http://www.satscan.org/)^11^. Since these are case counts, the discrete Poisson model was used^11^.

Spatial analysis is used to identify high or low risk clusters in space. This analysis does not consider the time factor. Spatiotemporal analysis, on the other hand, considers not only space, but also time as a whole. The parameters for running the tests are similar, only requiring the SaTScan to be informed of the test to be performed^11^.

To perform the scanning statistics, SaTScan received the following information: 1. number of cases, by municipality and year of occurrence, stratified by sex and age group; 2. population, by municipality and year, stratified by sex and age group; 3. geographic coordinates in latitude and longitude of the centroids of the municipalities^11^.

To ensure robust estimates, the Monte Carlo test was performed with 999 replications. To define the maximum size of the spatial cluster in relation to the population, the Gini coefficient, available in purely spatial scan statistics, was used to determine which non-overlapping spatial clusters should be reported. The optimal Gini coefficient (i.e., the largest possible value with p<0.05) suggests the maximum proportion of the total population that should be considered in the scan analyses^11^

In SaTScan, RR is the ratio between the incidence in the cluster and the incidence in the entire region, which was not included in the cluster. RR represents how much higher the risk of that disease is in this location and period compared to a baseline^11^.

The Prais-Winsten linear regression method was used in the temporal trend analyses, considering the years evaluated, 2001 to 2022. The municipal incidence rates of leptospirosis were transformed to the logarithmic scale (base 10)^12^. The results were expressed as annual percentage increase (API). The temporal trend was considered decreasing when the confidence interval (CI) values were negative, increasing when the values were positive and stationary when the confidence interval values included the value zero^12^. The adopted confidence interval was 95% (95%CI) and the significance level was 5%. The analyses were performed using the STATA 14 software.

The data used in this study are in the public domain and do not identify the individuals; therefore, submission to the Research Ethics Committee was waived.

## RESULTS

**Figure 1:**
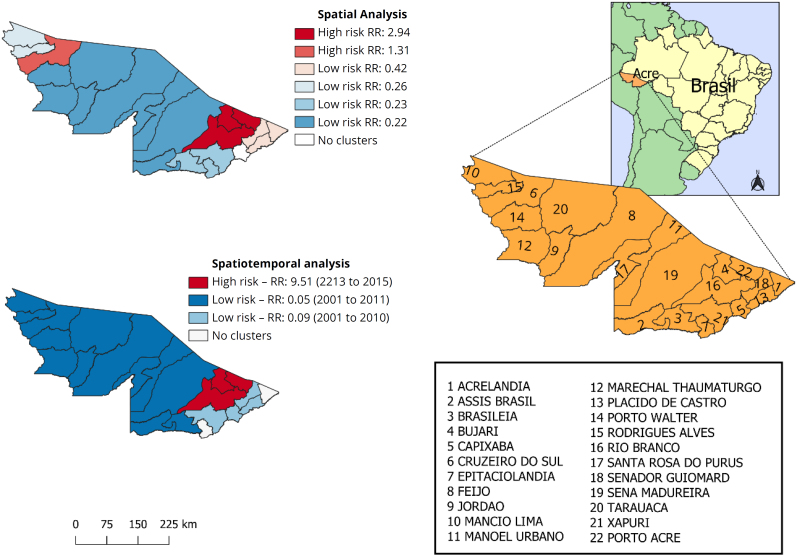
Spatial and spatiotemporal clusters of leptospirosis incidence, municipalities of the state of Acre, Brazil, 2001 to 2022.

Spatial analysis detected two high-risk clusters for leptospirosis in Acre between 2001 and 2022. The first (RR=2.94) was formed by the municipalities of Porto Acre (44.1/100,000 inh.), Rio Branco (43.6/100,000 inh.) and Bujari (32.5/100,000 inh.) and the second (RR=1.31), only by the municipality of Cruzeiro do Sul (36.6/100,000 inh.). The municipality of Capixaba was not related to any cluster (26.1/100,000 inh.). Other municipalities were detected as clusters of lower risk for the disease (RR=0.22∼0.42) (Figure 1). The spatiotemporal analysis once again showed the municipalities of Porto Acre, Rio Branco and Bujari as high-risk clusters for the disease, highlighting the period from 2013 to 2015 (Figure 1). Considering space and time simultaneously, the RR in the municipalities mentioned was 9.51 times that of the other locations. In 2014, the middle of the period, it reached incidences of 314.0/100,000 inh. in Porto Acre, 214.7/100,000 inh. in Rio Branco and 189.7/100,000 inh. in Bujari.

Spatiotemporal analysis also detected two low-risk clusters for the disease (RR=0.05∼0.09); however, these only occurred until the first decade of the study, between 2001 and 2011 (Figure 1).

The municipalities of Cruzeiro do Sul, Mâncio Lima, Marechal Thaumaturgo, Porto Walter and Rodrigues Alves (Table 1) showed an increasing temporal trend in the incidence of leptospirosis. It is worth noting that all of these municipalities are contiguous and are part of a microregion called Cruzeiro do Sul. The percentage of annual increase varied between 6 and 10%. Other municipalities in the state showed stable temporal trends. It is worth noting that no municipality showed a decreasing temporal trend in the incidence of the disease.

**Table 1:** Temporal trend of leptospirosis incidence, municipalities of the state of Acre, Brazil, 2001 to 2022.

Municipality	Incidence	Cases	%	API	95%CI	Interpretation
Acrelândia	17.49	51	1.04	0.05	-0.01;0.11	Stable
Assis Brasil	6.37	9	0.18	0.03	-0.01;0.07	Stable
Brasiléia	5.42	27	0.55	0.02	-0.02;0.06	Stable
Bujari	32.51	64	1.30	0.02	-0.06;0.09	Stable
Capixaba	26.17	54	1.10	0.05	-0.03;0.12	Stable
Cruzeiro do Sul	36.62	664	13.53	0.10	0.05-0.15	Growing
Epitaciolândia	3.13	11	0.22	0.02	-0.02;0.06	Stable
Feijó	12.34	92	1.87	0.06	-0.00;0.11	Stable
Jordão	4.56	7	0.14	0.02	-0.03;0.06	Stable
Mâncio Lima	9.59	34	0.69	0.08	0.05-0.11	Growing
Manoel Urbano	3.24	6	0.12	0.01	-0.03;0.5	Stable
Mal. Thaumaturgo	4.80	16	0.33	0.06	0.03-0.08	Growing
Plácido de Castro	10.05	40	0.81	0.03	-0.04;0.09	Stable
Porto Walter	6.52	14	0.29	0.06	0.03-0.10	Growing
Rio Branco	43.67	3411	69.48	0.04	-0.03;0.10	Stable
Rodrigues Alves	5.93	20	0.41	0.06	0.02-0.09	Growing
Sta. Rosa dos Purus	4.52	5	0.10	0.02	-0.00;0.05	Stable
Sen. Guiomard	12.00	56	1.14	0.04	-0.02;0.10	Stable
Sena Madureira	5.89	52	1.06	0.02	-0.03;0.07	Stable
Tarauacá	8.96	74	1.51	0.03	-0.01;0.8	Stable
Xapuri	13.11	49	1.00	0.06	-0.01;0.10	Stable
Porto Acre	44.14	153	3.12	0.04	-0.06;0.14	Stable

Incidence: cases/100,000 inh.; Cases: absolute frequency of cases in the period; %: relative frequency of cases in the period; API: annual percentage increase of cases; 95%CI: 95%.confidence interval

**Figure 2:**
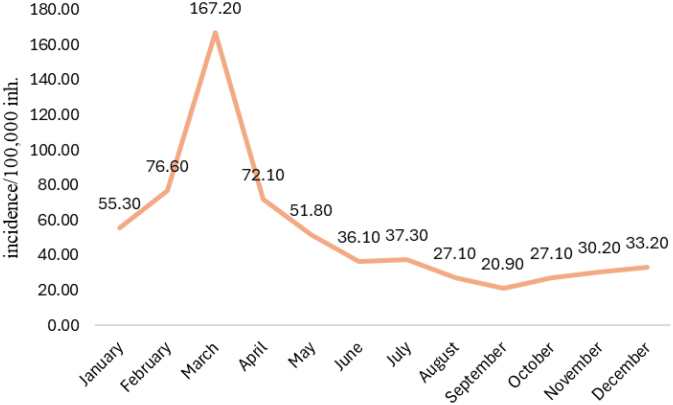
Monthly historical series of the incidence of leptospirosis in the state of Acre, Brazil, 2001 to 2022 (incidence/100,000 inh.).

The study observed that the incidence of leptospirosis in Acre occurred mainly in the first half of the year. Between 2001 and 2022, the incidence of leptospirosis in March (167.2/100,000 inh.) was 2.18 times higher than in February (76.6/100,000 inh.), the second month with the highest incidence (95%CI 1.98-2.40) (Figure 2).

**Figure 3:**
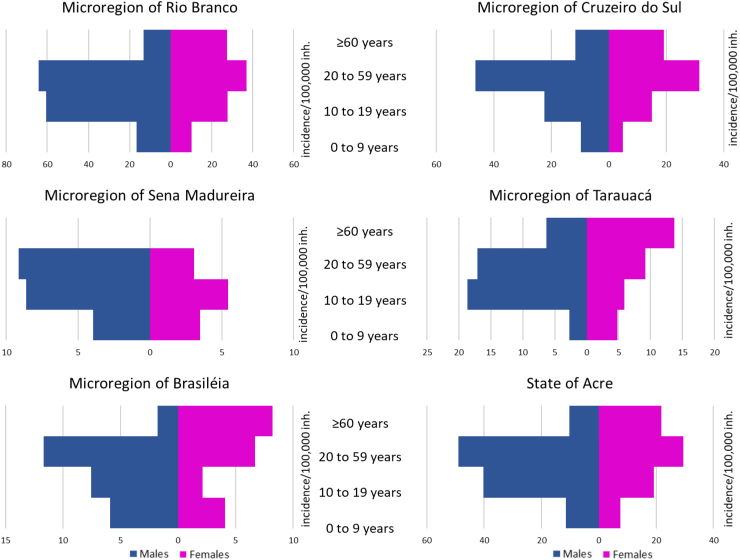
Epidemiological profile by sex and age group of the incidence of leptospirosis, microregions of the state of Acre, Brazil, 2001 to 2022.

The incidence of leptospirosis in Acre was higher in males of working age, 20 to 59 years old, followed by young people aged 10 to 19 years old (Figure 3). This was the pattern in the microregions of the state, especially in those with the highest incidence, Rio Branco and Cruzeiro do Sul. However, it is also worth noting that being an older woman was a risk factor for leptospirosis in the microregions of the state. In older women, RR of leptospirosis in Acre was 2.1 times higher than in older men (95%CI 1.6-2.9).

## DISCUSSION

High-risk spatial clusters for leptospirosis were identified in municipalities such as Porto Acre, Rio Branco, and Cruzeiro do Sul. These municipalities were established on the banks of rivers, which were the main routes for transportation, trade, and water supply at the time of their emergence. Over the years, there has been an increase in population density and disorderly urban growth, especially along the riverbanks^13-15^.

The municipalities of Rio Branco and Cruzeiro do Sul are the two most populous in Acre. In these locations, it is common to find disorderly urban growth, poor infrastructure, accumulation of garbage, presence of synanthropic animals, and frequent occurrence of floods^13,15^. These factors tend to contribute to the increased incidence of leptospirosis^14,16-18^.

The relationship between leptospirosis and floods has been known for a long time^19^. The emergence of leptospirosis is closely linked to exposure to ecological conditions that facilitate transmission^14^. In Rio Branco, in the last 49 years, there have been 37 small, medium, large, or extraordinary floods^13,20^.

A high-risk spatial-temporal cluster was identified in Porto Acre, Rio Branco and Bujari in the period from 2013 to 2015, with a RR for leptospirosis of 9.51 times compared to the other municipalities during this period. In these years, there were major floods, with emphasis on 2015, when the Acre River reached a historic mark, exceeding the overflow level by more than four meters^13,20^.

Of the state's floods, 73% occur in the months of February and March, which are the months of greatest rainfall, coinciding with the highest incidence of cases of the disease, as observed in this study^13,21^. Furthermore, during this period, the Amazonian pattern favors the survival of microorganisms due to the heavy rainfall and high temperatures^14^.

The municipality of Bujari was identified as a high-risk spatial and spatiotemporal cluster for leptospirosis, along with Porto Acre and Rio Branco. However, it is important to note that the municipality is not subject to flooding, as it does not have a river^22^. One hypothesis is that men of working age in Bujari had work or recreational contact with floods that occurred in Rio Branco between 2013 and 2015, since the municipalities are 25 km apart. However, to better understand the dynamics of leptospirosis transmission in Bujari, a more in-depth epidemiological investigation is needed, through interviews or by conducting a spatial study with the geolocation of leptospirosis cases^23^.

In addition to exposure to flooding, systematic reviews that considered leptospirosis and its risk factors demonstrated that transmission is significantly related to recreational activities in water, contact with mud and/or stagnant water, outdoor activities, and consumption of untreated water^17,24^.

Leptospirosis is endemic in all municipalities in Acre. Many of these municipalities are located on the banks of rivers and frequently experience flooding of varying degrees. However, it was observed that most municipalities in the state were identified as low-risk clusters for the disease and showed a stationary temporal trend, that is, the incidence of cases maintained a stable pattern.

It is important to emphasize that the magnitude of environmental risk factors varies according to geographic diversity, climate patterns, levels of urbanization, population growth and the socioeconomic status of the location in question^14,17^. In addition, investments in sanitary infrastructure, ensuring the supply of drinking water, implementing rodent control programs, and effective vaccination practices for livestock and pets are particularly important measures to mitigate the risk of the disease^17^.

The municipality of Cruzeiro do Sul and four neighboring municipalities recorded an increasing temporal trend in the incidence of leptospirosis during the period studied. These municipalities had a discrete or even non-existent incidence between 2001 and 2011. However, after this period, they were affected by floods of the Purus ^17^.and Juruá Rivers, which favored the increase in cases of leptospirosis in the region^20^.

Although there is no predisposition of sex or age to contract the infection, it was observed that, among the confirmed cases, males between 20 and 59 years old were among the most affected, followed by boys aged 10 to 19 years. These higher risk groups were similar in all microregions studied.

Morbidity in males is generally higher due to exposure to unhealthy work situations^1,25^. In addition, there are professions that provide greater contact with the bacteria that causes the disease, such as manual laborers, civil defense workers, military personnel, firefighters, street cleaners, garbage collectors, veterinarians, butchers, animal caretakers, fishermen, and farmers, among others^1,2^. Young people aged 10 to 19 can contract leptospirosis during recreational or work activities involving contaminated water^1^.

Older women had a higher RR than older men. One possible explanation would be that women become infected when performing domestic activities^26^.

The limitations of the study include the quality and reliability of secondary data, such as underreporting in municipalities with low incidence or overreporting, which is common during epidemics. In addition, the ecological study is subject to limitations, such as ecological bias, in which associations observed at the population level may not necessarily reflect associations at the individual level, and the inability to control confounding factors.

The findings demonstrated that leptospirosis, although endemic in the state, had a more significant incidence in certain municipalities and years. The study also showed that the Cruzeiro do Sul microregion showed an increasing trend in the incidence of leptospirosis in all of its five municipalities, while the other municipalities in the state had a stationary trend for the period.

Thus, it can be inferred from the findings that although the epidemiological profiles are similar in all microregions, it is not possible to have a single strategy against leptospirosis that is valid for the entire state. Therefore, it is necessary to act with greater or lesser intensity in certain places and periods, both for the prevention and control of the disease.

We also emphasize the importance of epidemiological surveillance using tools such as SaTScan, which through scanning statistics, can show where and when to concentrate greater efforts to carry out interventions.
