# Supplementary material for: Spatial, temporal, and space-time analysis of leptospirosis cases in Acre, 2001-2022
Source: Rev Bras Epidemiol. 2024 Nov 16;27:e240063. doi: 10.1590/1980-549720240063 (PMC11654289; doi:10.1590/1980-549720240063)
Supplement: Translation

# Translation: Análise espacial, temporal e espaçotemporal dos casos de leptospirose no Acre, 2001-2022

**DOI:** 10.1590/1980-549720240063.2

**Authors:** Leonardo Augusto Kohara Melchior, Kívia Roberta Costa da Silva, Ana Elisa Pereira Silva, Francisco Chiaravalloti-Neto

**Affiliations:** I Universidade Federal do Acre, Programa de Pós-Graduação em Ciências da Saúde na Amazônia Ocidental - Rio Branco (AC), Brasil.; II Universidade de São Paulo, Faculdade de Saúde Pública, Pós-Graduação em Saúde Pública - São Paulo (SP), Brasil.

**Keywords:** Análise espacial, Análise espaçotemporal, Ecossistema amazônico, Leptospira

## Abstract

**Objetivo:**

Identificar aglomerados de alto e baixo risco para a ocorrência da leptospirose no espaço e espaço-tempo do Acre, entre 2001 e 2022, bem como caracterizar tendências temporais e perfis epidemiológicos da doença no estado.

**Métodos:**

Estudo ecológico de casos notificados obrigatoriamente pelos serviços de saúde do Brasil. Para a análise de aglomerados no espaço e no espaço-tempo, utilizou-se o *software* SaTScan, que calculou os riscos relativos (RR). Além disso, foi obtida a tendência temporal pela regressão linear de Prais-Winsten, e os perfis epidemiológicos foram estimados pelas incidências por sexo e faixa etária.

**Resultados:**

Identificou-se aglomerado espacial de alto risco em Rio Branco, Bujari e Porto Acre (RR=2,94), ocorrendo sobretudo entre 2013 e 2015, conforme aglomerado espaçotemporal (RR=9,51). O município de Cruzeiro do Sul também apresentou aglomerado espacial de alto risco (RR=1,31). Esse município e outros contíguos apresentaram tendência temporal crescente nos casos, enquanto os demais municípios do estado mostraram tendência temporal estacionária. A doença afetou sobretudo homens entre 20 e 59 anos, seguidos por jovens de 10 a 19 anos. No entanto, o RR de leptospirose em mulheres idosas foi 2,1 vezes maior do que em homens idosos (intervalo de confiança de 95% [IC95%] 1,6-2,9).

**Conclusão:**

Os achados demonstraram que a leptospirose, embora endêmica no estado, teve incidência mais significativa em determinados municípios e anos. Logo, é necessário agir com maior ou menor intensidade em determinados locais e períodos, tanto para a prevenção quanto para o controle da enfermidade.

## INTRODUÇÃO

A leptospirose é uma doença cosmopolita infecciosa febril aguda, causada por bactérias do gênero *Leptospira* ^1^. É transmitida diretamente ou indiretamente de animais para seres humanos. Normalmente, a infecção ocorre pela exposição da pele e mucosas a água contaminada por urina de animais infectados, especialmente ratos^1^. A doença está fortemente associada a eventos hidrológicos, sobretudo em áreas tropicais e subtropicais com alta pluviosidade, bem como locais de urbanização precária e acúmulo de lixo^2^.

Embora a incidência por leptospirose no mundo não seja conhecida com precisão, estima-se que a doença seja uma das principais causas de morbidade por zoonoses no mundo^2^. No Brasil, entre 2001 e 2022, foram registrados 76.269 casos da doença, sendo esta endêmica em todos os estados^3^. O estado do Acre se destacou no período, pois sua incidência (29,67/100 mil hab.) foi 3,67 vezes superior à do segundo estado com maior incidência, o Amapá (8,08/100 mil hab.) e 8,46 vezes superior à do Brasil (3,51/100 mil hab.)^3^.

Alguns estudos científicos têm empregado técnicas de varredura para investigar se os casos de determinada doença apresentam uma distribuição aleatória no espaço, no tempo e no espaço-tempo^4-6^. Essas ferramentas têm possibilitado a identificação de aglomerados de casos, que são áreas críticas de transmissão da enfermidade. Com base nesses achados, é possível obter uma compreensão mais aprofundada da doença, o que contribui para a implementação de intervenções mais eficazes na prevenção e controle^7,8^.

Desta forma, para melhor compreensão da epidemiologia da leptospirose, este estudo propôs identificar aglomerados de alto e baixo risco para a ocorrência da doença no espaço e espaço-tempo no estado do Acre, entre 2001 e 2022. Complementarmente, buscou-se caracterizar as tendências temporais e os perfis epidemiológicos da leptospirose no estado.

## MÉTODOS

Estudo do tipo ecológico que utilizou o Acre como área de estudo. O Acre é um estado localizado ao norte do Brasil, na parte sudoeste da Amazônia Ocidental^9^. Possui área de 164.173 km^2^ e população estimada de 830.018 habitantes, com índice de desenvolvimento humano (IDH) de 0,71^9^. A economia do estado baseia-se, principalmente, no setor primário, apresentando um dos menores produtos internos brutos (PIB) do Brasil^9^.

O Acre é recoberto pela floresta Amazônica e apresenta clima quente e muito úmido do tipo Af e Am de Köppen. As temperaturas médias mensais variam entre 24 e 27°C e a pluviosidade atinge anualmente cerca de 2.100 mm. O estado possui uma estação chuvosa entre novembro e abril e uma estação seca entre maio e outubro^10^.

Para o estudo, as unidades espaciais foram os munícipios do estado do Acre, e as temporais, os anos de 2001 a 2022 e os meses de janeiro a dezembro.

Analisaram-se, neste estudo, os casos autóctones e confirmados de leptospirose do estado do Acre, entre 2001 e 2022. Foram considerados casos confirmados aqueles que atendiam ao critério de confirmação clínico-laboratorial (exame positivo para a doença) ou clínico-epidemiológico (história epidemiológica sugestiva nos 30 dias anteriores ao início dos sintomas, como exposição a enchentes, esgoto, lixo, atividades ocupacionais de risco e presença em área de alto risco para leptospirose)^3^.

Esses dados foram registrados pelo Sistema de Informação de Agravos de Notificação (SINAN) e disponibilizados eletronicamente pelo Departamento de Informática do Sistema Único de Saúde (DATASUS), Ministério da Saúde, Brasil^3^. Os casos foram extraídos por município, sexo (masculino e feminino) e faixa etária (0 a 9 anos, 10 a 19 anos, 20 a 59 anos, 60 anos ou superior).

Os dados demográficos de 2001 a 2022 foram obtidos do "Estudo de estimativas populacionais por município, sexo e idade, 2000 a 2022", disponibilizados, também, pelo DATASUS. Estes dados foram extraídos para o estado do Acre por sexo, faixa etária e por município^3^.

As taxas anuais de incidência de leptospirose foram calculadas pela razão entre o número de casos e a população do município, estratificadas por sexo e faixa etária e multiplicadas por 100 mil habitantes. A estratificação busca compreender a influência de variações por sexo e faixa etária na população, entre os municípios. O RR foi calculado pela razão entre as incidências.

Para facilitar a compreensão do perfil epidemiológico da leptospirose por faixa etária e sexo, os municípios foram agrupados em microrregiões geográficas, ou seja, municípios contíguos que apresentam especificidades. Assim, agruparam-se os 22 municípios em cinco microrregiões: Rio Branco (Acrelândia, Bujari, Capixaba, Plácido de Castro, Porto Acre, Rio Branco e Senador Guiomard), Sena Madureira (Manoel Urbano, Santa Rosa do Purus e Sena Madureira), Brasiléia (Assis Brasil, Brasiléia, Epitaciolândia e Xapuri), Cruzeiro do Sul (Cruzeiro do Sul, Mâncio Lima, Marechal Thaumaturgo, Porto Walter e Rodrigues Alves) e Tarauacá (Feijó, Jordão e Tarauacá)^9^.

Para a identificação de aglomerados de alto e baixo risco de leptospirose, no espaço e no espaço-tempo no estado do Acre, utilizaram-se estatísticas puramente espaciais e espaçotemporais usando o *software* SaTScan, versão 10.1.1 (http://www.satscan.org/)^11^. Por tratar-se de contagens de casos, utilizou-se o modelo discreto de Poisson^11^.

A análise espacial serve para identificar aglomerados de alto ou baixo risco no espaço. Essa análise não considera o fator tempo. Já a análise espaçotemporal considera não apenas o espaço, mas também o tempo em conjunto. Os parâmetros para rodar os testes são similares, apenas necessitando-se informar ao SaTScan o teste a ser realizado^11^.

Para realizar as estatísticas de varredura, o SaTScan recebeu as seguintes informações: 1. quantidade de casos, por município e ano de ocorrência, estratificadas por sexo e faixa etária; 2. população, por município e ano, estratificada por sexo e faixa etária; 3. coordenadas geográficas em latitude e longitude dos centroides dos municípios^11^.

Para garantir estimativas robustas, o teste de Monte Carlo foi realizado com 999 replicações. Para definir o tamanho máximo do *cluster* espacial em relação à população, o coeficiente de Gini, disponível na estatística de varredura puramente espacial, foi usado para determinar quais *clusters* espaciais não sobrepostos deveriam ser relatados. O coeficiente de Gini ótimo (ou seja, o maior valor possível com p<0,05) sugere qual a proporção máxima da população total que deve ser considerada nas análises de varredura^11^.

No SaTScan, o RR é a razão entre a incidência no aglomerado e a incidência em toda a região, que não foi incluída no aglomerado. O RR representa o quanto o risco dessa doença é maior neste local e período em comparação com uma linha de base^11^.

O método de regressão linear de Prais-Winsten foi utilizado nas análises de tendência temporal, considerando-se os anos avaliados, 2001 a 2022. As taxas de incidência municipal de leptospirose foram transformadas para a escala logarítmica (base 10)^12^. Os resultados foram expressos em incremento percentual anual (IPA). A tendência temporal foi considerada decrescente quando os valores do intervalo de confiança (IC) eram negativos; crescente, quando os valores eram positivos; e estacionária, quando os valores do intervalo de confiança incluíam o valor zero^12^. O intervalo de confiança adotado foi de 95% (IC95%) e o nível de significância de 5%. As análises foram realizadas com o uso do *software* STATA 14.

Os dados utilizados neste estudo são de domínio público e sem identificação dos indivíduos, portanto é dispensada a submissão ao Comitê de Ética em Pesquisa.

## RESULTADOS

A análise espacial detectou dois aglomerados de alto risco para leptospirose no Acre no período entre 2001 e 2022. O primeiro (RR=2,94) formado pelos municípios de Porto Acre (44,1/100 mil hab.), Rio Branco (43,6/100 mil hab.) e Bujari (32,5/100 mil hab.) e o segundo (RR=1,31), somente pelo município de Cruzeiro do Sul (36,6/100 mil hab.). O município de Capixaba não foi relacionado a nenhum aglomerado (26,1/100 mil hab.). Demais municípios foram detectados como aglomerados de menor risco para a doença (RR=0,22∼0,42) (Figura 1).

**Figura 1:** Aglomerados espaciais e espaçotemporais das incidências de leptospirose, municípios do estado do Acre, Brasil, 2001 a 2022.

A análise espaçotemporal apresentou novamente os munícipios de Porto Acre, Rio Branco e Bujari como aglomerados de alto risco para a doença, destacando o período de 2013 a 2015 (Figura 1). Considerando o espaço e tempo simultaneamente, o RR nos municípios citados foi de 9,51 vezes o das demais localidades. Atingiu em 2014, meio do período, incidências de 314,0/100 mil hab. em Porto Acre, 214,7/100 mil hab. em Rio Branco e 189,7/100 mil hab. em Bujari.

A análise espaçotemporal também detectou dois aglomerados de baixo risco para a doença (RR=0,05∼0,09), entretanto estes ocorreram somente até a primeira década do estudo, entre 2001 e 2011 (Figura 1).

Apresentaram tendência temporal crescente da incidência de leptospirose os municípios de Cruzeiro do Sul, Mâncio Lima, Marechal Thaumaturgo, Porto Walter e Rodrigues Alves (Tabela 1). Ressalta-se que todos esses municípios são contíguos e fazem parte de uma microrregião chamada de Cruzeiro do Sul. A porcentagem de incremento anual (PIA) variou entre 6 e 10%. Demais municípios do estado apresentaram tendências temporais estáveis. Ressalta-se que nenhum município apresentou tendência temporal decrescente de incidência da doença.

**Tabela 1:** Tendência temporal da incidência de leptospirose, munícipios do estado do Acre, Brasil, 2001 a 2022.

Municípios	Incidência	Casos	%	PIA	IC95%	Interpretação
Acrelândia	17,49	51	1,04	0,05	-0,01;0,11	Estável
Assis Brasil	6,37	9	0,18	0,03	-0,01;0,07	Estável
Brasiléia	5,42	27	0,55	0,02	-0,02;0,06	Estável
Bujari	32,51	64	1,30	0,02	-0,06;0,09	Estável
Capixaba	26,17	54	1,10	0,05	-0,03;0,12	Estável
Cruzeiro do Sul	36,62	664	13,53	0,10	0,05-0,15	Crescente
Epitaciolândia	3,13	11	0,22	0,02	-0,02;0,06	Estável
Feijó	12,34	92	1,87	0,06	-0,00;0,11	Estável
Jordão	4,56	7	0,14	0,02	-0,03;0,06	Estável
Mâncio Lima	9,59	34	0,69	0,08	0,05-0,11	Crescente
Manoel Urbano	3,24	6	0,12	0,01	-0,03;0,5	Estável
Mal. Thaumaturgo	4,80	16	0,33	0,06	0,03-0,08	Crescente
Plácido de Castro	10,05	40	0,81	0,03	-0,04;0,09	Estável
Porto Walter	6,52	14	0,29	0,06	0,03-0,10	Crescente
Rio Branco	43,67	3411	69,48	0,04	-0,03;0,10	Estável
Rodrigues Alves	5,93	20	0,41	0,06	0,02-0,09	Crescente
Sta. Rosa dos Purus	4,52	5	0,10	0,02	-0,00;0,05	Estável
Sen. Guiomard	12,00	56	1,14	0,04	-0,02;0,10	Estável
Sena Madureira	5,89	52	1,06	0,02	-0,03;0,07	Estável
Tarauacá	8,96	74	1,51	0,03	-0,01;0,8	Estável
Xapuri	13,11	49	1,00	0,06	-0,01;0,10	Estável
Porto Acre	44,14	153	3,12	0,04	-0,06;0,14	Estável

Incidência: casos/100 mil habitantes; Casos: frequência absoluta de casos no período; %: frequência relativa de casos no período; PIA: porcentagem de incremento anual do número de casos; IC95%: intervalo de confiança de 95%.

O estudo observou que a incidência de leptospirose no Acre ocorreu principalmente no primeiro semestre do ano. Entre 2001 e 2022, a incidência da leptospirose em março (167,2/100 mil hab.) foi 2,18 vezes maior que em fevereiro (76,6/100 mil hab.), segundo mês de maior incidência (IC95% 1,98-2,40) (Figura 2).

**Figura 2:** Série histórica mensal da incidência da leptospirose no estado do Acre, Brasil, 2001 a 2022 (incidência/100 mil hab.).

A incidência de leptospirose no Acre foi maior em indivíduos do sexo masculino na idade produtiva de 20 a 59 anos, seguidos por jovens de 10 a 19 anos (Figura 3). Este foi o padrão nas microrregiões do estado, sobretudo naquelas de maior incidência, Rio Branco e Cruzeiro do Sul. Contudo, destaca-se ainda que ser mulher idosa foi fator de risco para leptospirose nas microrregiões do estado. Em mulheres idosas, o RR de leptospirose no Acre foi 2,1 maior que em homens idosos (IC95% 1,6-2,9).

**Figura 3:** Perfil epidemiológico por sexo e faixa etária da incidência de leptospirose, microrregiões do estado do Acre, Brasil, 2001 a 2022.

## DISCUSSÃO

Os aglomerados espaciais de alto risco para leptospirose foram identificados em municípios como Porto Acre, Rio Branco e Cruzeiro do Sul. Esses municípios foram estabelecidos às margens de rios, que eram as principais vias de transporte, comércio e abastecimento de água na época de seu surgimento. Com o passar dos anos, houve aumento na densidade populacional e crescimento urbano desordenado, especialmente ao longo das margens dos rios^13-15^.

Os municípios de Rio Branco e Cruzeiro do Sul são os dois mais populosos do Acre. Nessas localidades, é comum encontrar crescimento urbano desordenado, infraestrutura precária, acúmulo de lixo, presença de animais sinantrópicos e ocorrência frequente de enchentes^13,15^. Esses fatores tendem a contribuir para o aumento da incidência de leptospirose^14,16-18^.

A relação entre leptospirose e enchentes é conhecida há bastante tempo^19^. O surgimento da leptospirose está intimamente ligado à exposição a condições ecológicas que facilitam a transmissão^14^. Em Rio Branco, entre últimos 49 anos, em 37 ocorreram enchentes de porte pequeno, médio, grande ou extraordinário^13,20^.

Identificou-se um aglomerado espaçotemporal de alto risco em Porto Acre, Rio Branco e Bujari no período de 2013 a 2015, apresentando RR para leptospirose de 9,51 vezes em comparação com os demais municípios durante esse período. Nesses anos, houve grandes enchentes, com destaque para 2015, quando o rio Acre atingiu uma marca histórica, ultrapassando em mais de quatro metros a cota de transbordamento^13,20^.

Das enchentes do estado, 73% ocorrem nos meses de fevereiro e março, que são os de maiores precipitações, coincidindo com a maior incidência dos casos da doença, conforme observado neste estudo^13,21^. Além disso, nesse período, o padrão amazônico favorece a sobrevivência de microrganismos em razão da elevada pluviosidade e temperaturas^14^.

O município de Bujari foi identificado como aglomerado espacial e espaçotemporal de alto risco para leptospirose, junto com Porto Acre e Rio Branco. No entanto, é importante salientar que o município não está sujeito a enchentes, pois não possui rio^22^. Uma hipótese é que homens na idade produtiva de Bujari tenham tido contato laboral ou recreativo com enchentes que ocorreram em Rio Branco entre 2013 e 2015, visto que os municípios distam 25 km. No entanto, para entender melhor a dinâmica de transmissão da leptospirose em Bujari, é necessária uma investigation epidemiológica mais aprofundada, por meio de entrevistas ou da condução de um estudo espacial com a geolocalização dos casos de leptospirose^23^.

Além da exposição a enchentes, as revisões sistemáticas que consideraram a leptospirose e seus fatores de risco demonstraram que a transmissão está significativamente relacionada a atividades recreativas na água, ao contato com lama e/ou água estagnada, a atividades ao ar livre e ao consumo de água não tratada^17,24^.

A leptospirose é endêmica em todos os municípios do Acre. Muitos desses municípios estão situados às margens de rios e enfrentam enchentes de diversos níveis com frequência. No entanto, observou-se que a maioria dos municípios do estado foi identificada como aglomerados de baixo risco para a doença e apresenta tendência temporal estacionária, ou seja, a incidência de casos manteve padrão estável.

É importante ressaltar que a magnitude dos fatores de risco ambientais varia de acordo com a diversidade geográfica, os padrões climáticos, os níveis de urbanização, o crescimento populacional e o *status* socioeconômico do local em questão^14,17^. Além disso, investimentos em infraestrutura sanitária, garantia de fornecimento de água potável, implementação de programas de controle de roedores e práticas de vacinação eficazes para o gado e animais de estimação são medidas particularmente importantes para mitigar o risco da doença^17^.

O município de Cruzeiro do Sul e quatro municípios vizinhos registraram tendência temporal crescente na incidência de leptospirose no período estudado. Esses municípios tinham uma incidência discreta ou até mesmo nula entre 2001 e 2011. No entanto, após esse período, foram afetados por enchentes dos rios Purus e Juruá, o que favoreceu o aumento dos casos de leptospirose na região^20^.

Embora não exista uma predisposição de sexo ou de idade para contrair a infecção, observou-se que, entre os casos confirmados, o sexo masculino com faixa etária entre 20 e 59 anos está entre os mais atingidos, seguido por rapazes de 10 a 19 anos. Esses grupos de maior risco foram semelhantes em todas as microrregiões do estudo.

A morbidade no sexo masculino geralmente é maior por conta da exposição a situações insalubres de trabalho^1,25^. Além disso, há profissões que propiciam maior contato com a bactéria causadora da doença, como trabalhadores braçais, funcionários da defesa civil, militares, bombeiros, garis, catadores de lixo, médicos veterinários, magarefes, tratadores de animais, pescadores, agricultores, entre outros^1,2^. Os jovens de 10 a 19 anos podem contrair leptospirose em atividades recreativas ou laborais com águas contaminadas^1^.

As mulheres idosas apresentaram RR maior do que homens idosos. Uma possível explicação seria que as mulheres se contaminam ao desempenharem atividades domésticas^26^.

As limitações do estudo incluem a qualidade e confiabilidade dos dados secundários, como a subnotificação em municípios com baixa incidência ou a supernotificação, comum durante epidemias. Além disso, o estudo ecológico está sujeito a limitações, como o viés ecológico, no qual associações observadas em nível populacional podem não refletir necessariamente associações em nível individual e a incapacidade de controlar fatores de confusão.

Os achados demonstraram que a leptospirose, embora endêmica no estado, teve incidência mais significativa em determinados municípios e anos. O estudo mostrou ainda que a microrregião de Cruzeiro do Sul apresentou tendência crescente da incidência de leptospirose em todos os seus cinco municípios, enquanto os demais municípios do estado tiveram tendência estacionária para o período.

Dessa forma, pode-se inferir pelos achados que, embora os perfis epidemiológicos sejam semelhantes em todas as microrregiões, não é possível ter uma estratégia única contra a leptospirose válida para todo o território estadual. Assim, é necessário agir com maior ou menor intensidade em determinados locais e períodos, tanto para a prevenção quanto para o controle da enfermidade.

Ressaltamos ainda a importância de a vigilância epidemiológica utilizar ferramentas tais como o SaTScan, que, por meio da estatística de varredura, consegue mostrar onde e quando concentrar maiores esforços para realizar intervenções.
